# Pronounced Photovoltaic Response from Multi-layered MoTe_2_ Phototransistor with Asymmetric Contact Form

**DOI:** 10.1186/s11671-017-2373-5

**Published:** 2017-11-22

**Authors:** Junku Liu, Nan Guo, Xiaoyang Xiao, Kenan Zhang, Yi Jia, Shuyun Zhou, Yang Wu, Qunqing Li, Lin Xiao

**Affiliations:** 1 Nanophotonics and Optoelectronics Research Center, Qian Xuesen Laboratory of Space Technology, China Academy of Space Technology, Beijing, 100094 China; 2 State Key Laboratory of Low-Dimensional Quantum Physics, Department of Physics and Tsinghua-Foxconn Nanotechnology Research Center, Tsinghua University, Beijing, 100084 China; 3 Department of Physics, Tsinghua University, Beijing, 100084 China

**Keywords:** MoTe_2_, Photovoltaic, Interface, Asymmetric

## Abstract

In this study, we fabricate air-stable p-type multi-layered MoTe_2_ phototransistor using Au as electrodes, which shows pronounced photovoltaic response in off-state with asymmetric contact form. By analyzing the spatially resolved photoresponse using scanning photocurrent microscopy, we found that the potential steps are formed in the vicinity of the electrodes/MoTe_2_ interface due to the doping of the MoTe_2_ by the metal contacts. The potential step dominates the separation of photoexcited electron-hole pairs in short-circuit condition or with small *V*_sd_ biased. Based on these findings, we infer that the asymmetric contact cross-section between MoTe_2_-source and MoTe_2_-drain electrodes is the reason to form non-zero net current and photovoltaic response. Furthermore, MoTe_2_ phototransistor shows a faster response in short-circuit condition than that with higher biased *V*_sd_ within sub-millisecond, and its spectral range can be extended to the infrared end of 1550 nm.

**Electronic supplementary material:**

The online version of this article (10.1186/s11671-017-2373-5) contains supplementary material, which is available to authorized users.

## Background

Graphene and similar two-dimensional (2D) materials exist in bulk form as stacks of strongly bonded layers with weak interlayer attraction, allowing itself to be exfoliated into individual, atomically thin layers, which have opened up new possibilities for the exploration of 2D physics as well as that of new material applications [1–9]. Of them, semiconductor transition metal dichalcogenides (TMDs) with the common formula MX_2_, where M stands for a transition metal from group VI (M = Mo, W) and X for a chalcogen element (S, Se, Te), exhibit sizeable bandgaps [2, 3, 10, 11]. In addition, these 2D TMD flakes are flexible and free of dangling bonds between adjacent layers [12, 13]. These unique properties make TMDs promising candidates to construct electronic and optoelectronic devices [2–4, 14–17], such as a next-generation field-effect transistor (FET) at sub-10 nm [18], on-chip light-emitting diode [19–21], and Van der Waals heterostructure devices [4, 5].

2H-type molybdenum ditelluride (2H-MoTe_2_) is one of the typical 2D TMDs, which has an indirect bandgap of 0.83 eV in bulk form [22] and a direct bandgap of 1.1 eV when it is thinned to monolayer [23]. 2H-MoTe_2_ has been explored for applications in spintronics [24], FET [25–27], photodetector [28–32], and solar cell [33]. Like most 2D materials, electrical metal contacts with 2H-MoTe_2_ play an important role in realizing high-performance electronic and optoelectronic devices. It has been proven that p-type and n-type contact doping and ohm contact can be realized using suitable contact materials [34–40], and they can, in turn, be used to construct functional devices, such as photovoltaic photodetector [37, 38] and diode [37]. Up to now, the research focus has been concentrated on evaluating and studying metal-semiconductor contacts by comparing various electrode materials, but insufficient attention has been paid to comparing metal-semiconducting contact forms in-depth, for example, the same contact material with asymmetric contact cross-section.

In this study, we fabricate air-stable p-type multi-layered MoTe_2_ phototransistor with asymmetric contact cross-section between MoTe_2_-source and MoTe_2_-drain electrodes and investigate its photoresponse using scanning photocurrent at different gate- and source-drain voltages. This study helps to reveal the spatial potential profiles and analyze the impact of contact in the device. Experimental data show that the device has non-zero net photocurrent in short-circuit condition and photovoltaic response. Scanning photocurrent map reveals that strong photocurrent is generated in the vicinity of contact interface in short-circuit condition or with small source-drain voltage (*V*_sd_) biased, which indicates the potential steps are formed in the vicinity of the electrodes/MoTe_2_ interface due to the doping of the MoTe_2_ by the metal contacts. When biased voltage *V*_sd_ rises above the potential step, *V*_sd_ dominates the separation of photoexcited electron-hole pairs and photocurrent (*I*_PC_ *= I*_sd_ *− I*_dark_) peak appears in the center of the device channel. This indicates the asymmetric contact cross-section between MoTe_2_-source and MoTe_2_-drain electrodes is the reason to form non-zero net current and photovoltaic response. This finding is helpful to construct photovoltaic photodetector with low power consumption. Finally, we test the time-resolved and wavelength-dependent photocurrent of MoTe_2_ phototransistor, obtaining sub-millisecond response time and finding that its spectral range can be extended to the infrared end of 1550 nm.

## Results and Discussion

**Fig. 1:**
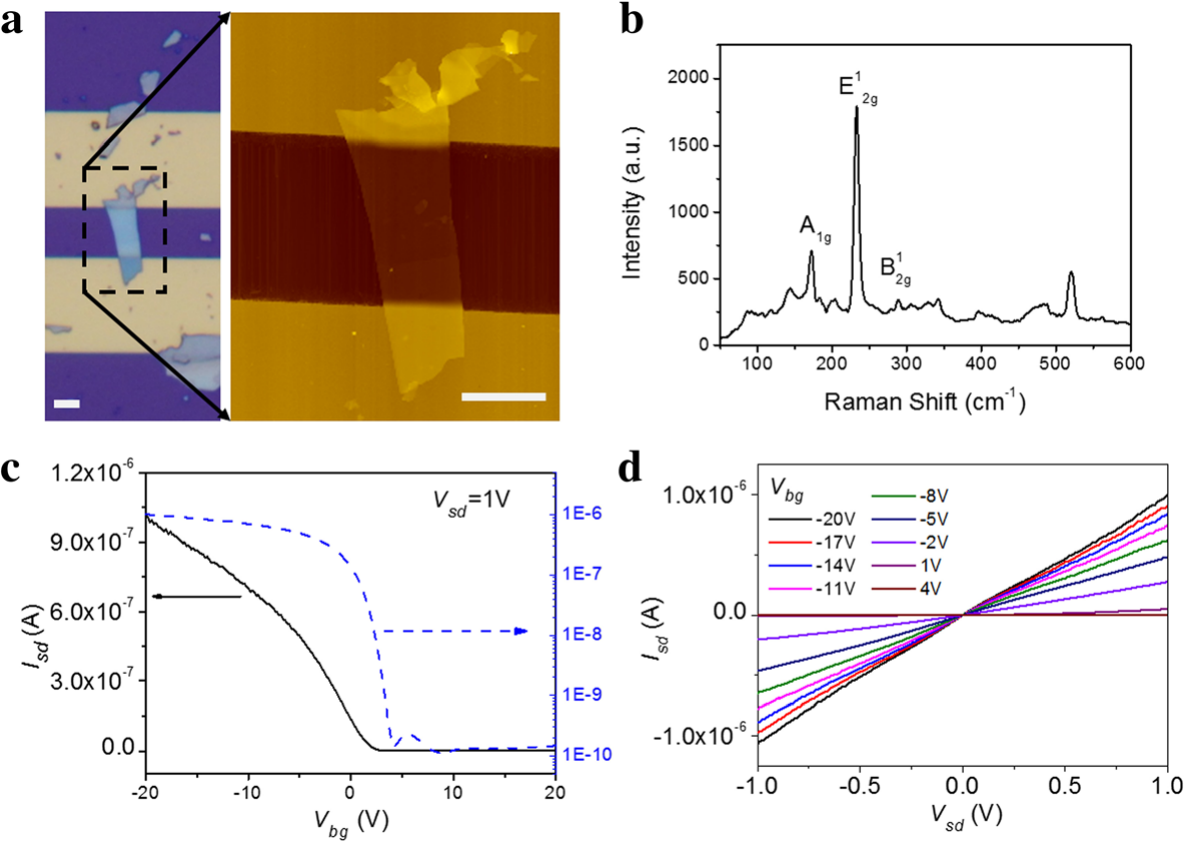
**a** Optical image and AFM image of multi-layered MoTe_2_ phototransistor. The scale bars are 5 μm. **b** Raman spectrum of multi-layered MoTe_2_ phototransistor with 514-nm laser excitation. **c** Transfer characteristics and **d** output characteristics of multi-layered MoTe_2_ phototransistor

We fabricate two back-gated multi-layered MoTe_2_ phototransistors (D1 and D2) and measure their photoresponse. The device is identified by an optical microscope, and the corresponding MoTe_2_ thickness and quality are characterized using atomic force microscopy (AFM) and Raman spectrum. All measurements are conducted in ambient condition. Figure 1a shows the optical image (left) and AFM image (right) of D1 (D2 is shown in Additional file 1: Figure S1. The following data are collected from D1 unless otherwise specified, and the data from D2 are shown in Additional file 1). The device consists of source electrode, drain electrode, and channel sample of multi-layered MoTe_2_ on SiO_2_/p^+^-Si substrate. SiO_2_ film with the thickness of 300 nm is dielectric, and p^+^-Si works as a back-gate electrode. The details of D1 are characterized using AFM, which shows that multi-layered MoTe_2_ straddles source and drain electrodes. The channel length is 10 μm. MoTe_2_ sample in the channel is about 23 nm thick (height profile is shown in Additional file 1: Figure S2), and the widths of MoTe_2_-source and MoTe_2_-drain contact cross-section are 6.5 and 4.8 μm, respectively. Figure 1b shows the Raman spectrum of MoTe_2_ sample. The characteristics Raman-active modes of A_1g_ (172 cm^−1^), E^1^_2g_ (233 cm^−1^), and B^1^_2g_ (289 cm^−1^) are clearly observed, confirming the good quality of MoTe_2_ in the channel.

Electric measurement indicates that multi-layered MoTe_2_ phototransistor is p-type as shown in Fig. 1c, which is in on-state at negative gate voltage and in off-state at positive gate voltage. The current on-off ratio is 6.8 × 10^3^ when source-drain voltage *V*_sd_ is 1 V. The field-effect mobility (μ) is 14.8 cm^2^/V s according to transfer characteristics. When biased voltage *V*_sd_ decreases from 1 V to 100 mV, on-current and off-current both decrease, and the on-off ratio is still above 6.0 × 10^3^, as shown in Additional file 1: Figure S3(a) and (b). When the gate voltage is swept from − 20 to 20 V and then back to − 20 V, multi-layered MoTe_2_ phototransistor shows small hysteresis (see Additional file 1: Figure S3(c)) and air-stable p-type conductance, which benefits from the simple fabrication process and polymer-free MoTe_2_ sample. We also fabricate other multi-layered MoTe_2_ phototransistor with a thickness of 5, 10, 11, 12, 15.7, and 38 nm, respectively, as shown in Additional file 1: Figure S4. They all show air-stable p-type conductance. Figure 1d shows the output characteristics of multi-layered MoTe_2_ transistor as back-gate voltage (*V*_bg_) varies from − 20 to 4 V. As seen, the response is essentially linear, especially at a low biased voltage of *V*_sd_, which indicates that there is a low Schottky barrier between Au and MoTe_2_ in the air.

**Fig. 2:**
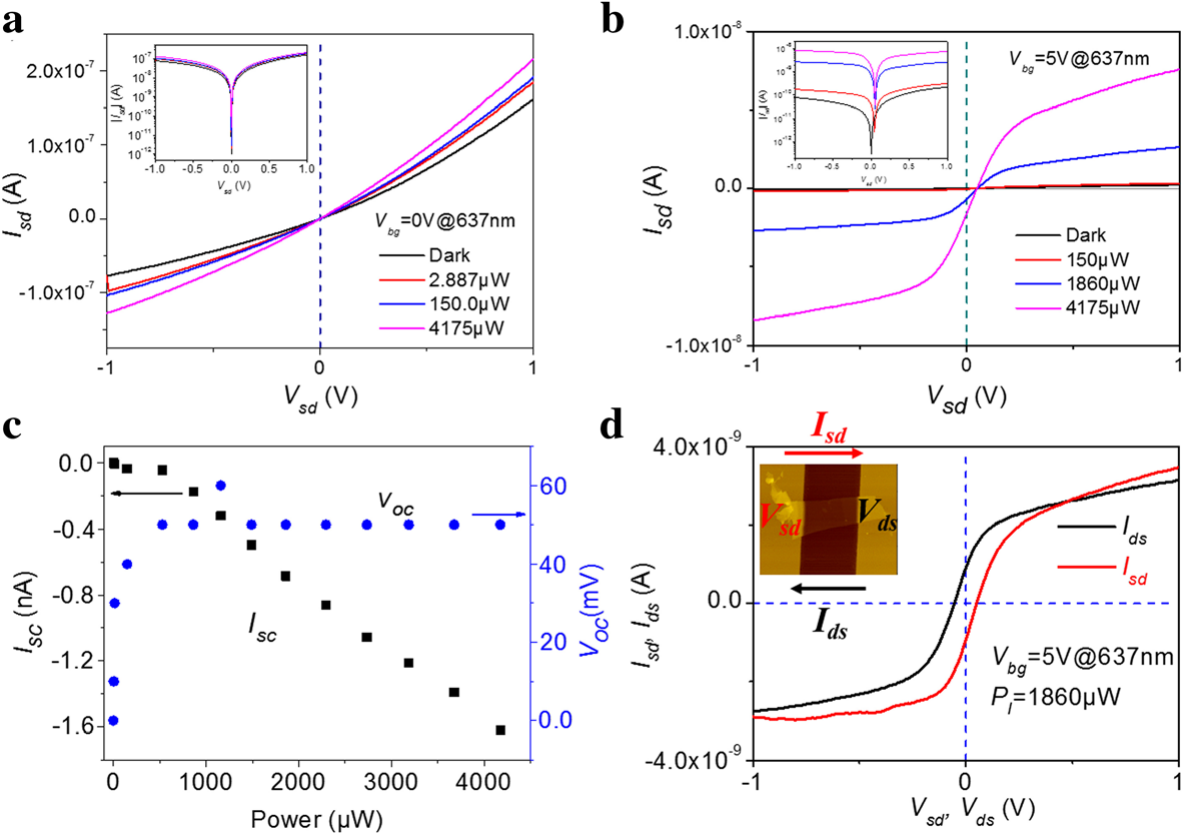
Photoresponse of multi-layered MoTe_2_ phototransistor illuminated by 637-nm wavelength laser in ambient condition. **a** *I*_sd_ vs. *V*_sd_ curves at *V*_bg_ = 0 V as illumination power increases. **b** *I*_sd_ vs. *V*_sd_ curves at *V*_bg_ = 5 V as illumination power increases. **c** *V*_OC_ and *I*_SC_ as a function of illumination power. **d** Output current for biased voltage loaded on the source and drain electrode, respectively

Figure 2 shows the photoresponse of multi-layered MoTe_2_ phototransistor when it is illuminated by 637-nm continuous-wave laser in ambient condition, which is conducted by combining Agilent B1500A semiconductor analyzer with Lakeshore probe station. Laser spot size is larger than 200 μm in diameter, and the device is covered with uniform illumination intensity. Backgate-dependent and power-dependent photoresponse are shown in Additional file 1: Figure S5. As shown in Fig. 2a, when a back-gate voltage is 0 V, source-drain current (*I*_sd_) increases with laser power. *I*_sd_ vs. *V*_sd_ curves at different illumination power levels all meet at *V*_sd_ = 0 V, which is clearly observed in a logarithmic plot of |*I*_sd_| shown in insert Figure of Fig. 2a. When *V*_bg_ = 5 V, the phototransistor is in off-state (see Fig. 1c), and the current of *I*_sd_ increases with the illumination laser power, exhibiting clear nonlinear behavior, as shown in Fig. 2b. Furthermore, the phototransistor shows non-zero open-circuit voltage (*V*_OC_) and short-circuit current (*I*_SC_) with laser illumination, which is the evidence of photovoltaic response from multi-layered MoTe_2_ phototransistor. Figure 2c shows *V*_OC_ and *I*_SC_ as a function of illumination power. *V*_OC_ remains unchanged at 50 mV (illumination power is higher than 500 μW), and |*I*_SC_| increases from 0 to 1.6 nA when laser power increases from 0 to 4175 μW. When we change the voltage direction, *V*_OC_ and *I*_SC_ remain unchanged as shown in Fig. 2d. *V*_sd_ represents the voltage loaded on source electrode and *V*_ds_ is loaded on drain electrode, and the corresponding current is indicated by *I*_sd_ and *I*_ds_, respectively. Insert image in Fig. 2d illustrates the voltage and current direction. Whether the voltage is loaded on the source or drain electrode, the *V*_OC_ of 50 mV relative to source voltage and corresponding *I*_SC_ of 680 pA flowing from drain electrode to source electrode both remain unchanged. This confirms the photovoltaic response of multi-layered MoTe_2_ phototransistor.

In order to reveal the mechanism of photoresponse, especially the photovoltaic response, we perform a scanning photocurrent microscopy (SPCM) study, which helps to obtain the spatial potential profiles and to analyze the spatially resolved photoresponse. SPCM is performed using a home-made scanning photocurrent setup in ambient condition. Optical excitation is provided by SuperK EXTREME supercontinuum white light laser. Its wavelength ranges from 400 to 2400 nm. The beam, with adjustable wavelength using SuperK SELECT multi-line tunable filter, is focused on the device using a 20× objective lens. A galvanometer mirror positioning system is used to make the laser beam scan the device to obtain photocurrent maps. The reflected light and the photocurrent are recorded with a current preamplifier and a lock-in amplifier at chopper frequency of 1 KHz.

**Fig. 3:**
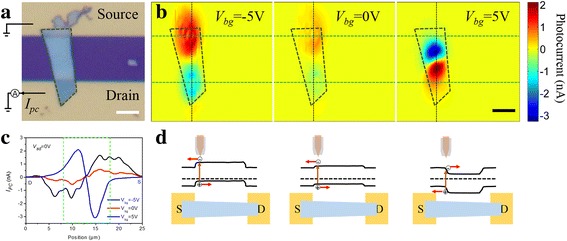
Spatial-resolved photocurrent images of D1 as a function of gate voltage. **a** The optical image together with the electrical setup. **b** Spatial-resolved photocurrent images at *V*_bg_ = − 5, 0, and 5 V, respectively. **c** *I*_PC_ profile collected from the black dashed line in Fig. 3b. **d** Corresponding potential profiles at *V*_bg_ = − 5, 0, and 5 V, respectively. The scale bars are 5 μm in all figures

Figure 3 shows the scanning photocurrent of D1 with an excitation wavelength of 1200 nm. Laser spot diameter is about 4.4 μm derived from the reflection image (see Additional file 1: Figure S7). Figure 3a shows the optical image, together with the electrical setup. *I*_PC_ measurements are conducted in short-circuit condition, in which source electrode is grounded and *I*_PC_ is collected from drain electrode. The current flowing from the source to drain electrode is positive. Figure 3b shows spatial-resolved photocurrent image collected at the gate voltage (*V*_bg_) of − 5, 0, and 5 V, respectively. It can be seen that short-circuit *I*_PC_ with opposite polarities is strong in the vicinity of the interfaces between MoTe_2_ and the electrodes. When *V*_bg_ is changed from − 5 to 0 V, *I*_PC_ pattern remains unchanged but the intensity decreases. *V*_bg_ is further increased to 5 V; *I*_PC_ not only switches polarity, the position of maximum *I*_PC_ also moves away from contact interface and into the channel. Figure 3c shows the *I*_PC_ profile taken from the black dashed line in Fig. 3b at *V*_bg_ = − 5, 0, and 5 V, respectively. It clearly demonstrates that *I*_PC_ has a broad intensity peak near the interface between MoTe_2_ and electrodes at *V*_bg_ = − 5 and 0 V, while the peak moves into the channel, which is about 3 μm away from the contact interface and becomes narrower.

The presence of *I*_PC_ peaks indicates the existence of potential steps in short-circuit condition. According to the *I*_PC_ distribution, we plot the corresponding potential profile along the device channel as shown in Fig. 3d. At *V*_bg_ = − 5 and 0 V, the potential steps are near the contact interface between MoTe_2_ and electrodes, and they move into the channel at *V*_bg_ = 5 V. According to the previous study [41], Au electrode contact introduces p-doping and pins the Fermi level of MoTe_2_ at contact part. Thus, the potential steps are formed in the vicinity of the electrode/MoTe_2_ interface as the Fermi level in the channel is modulated by the gate voltage. At *V*_bg_ = 0 V, a weak *I*_PC_ is observed, which flows from the electrode to MoTe_2_ channel. It means photoexcited electrons drift to nearby electrode and holes to MoTe_2_ channel. At *V*_bg_ = − 5 V, the hole density in MoTe_2_ channel is enhanced and induces a larger potential step in the vicinity of the electrode/MoTe_2_ interface. Photoexcited electron-hole pairs can be separated effectively and *I*_PC_ increases. When *V*_bg_ = 5 V, more electrons are injected into the MoTe_2_ channel, and potential well is formed in the channel. Because of electrostatics of electrode, the potential steps move away from the electrode and appear in the channel. The photoexcited electrons drift to the MoTe_2_ channel and holes toward the nearby electrode. *I*_PC_ changes direction compared with that at *V*_bg_ = − 5 and 0 V.

**Fig. 4:**
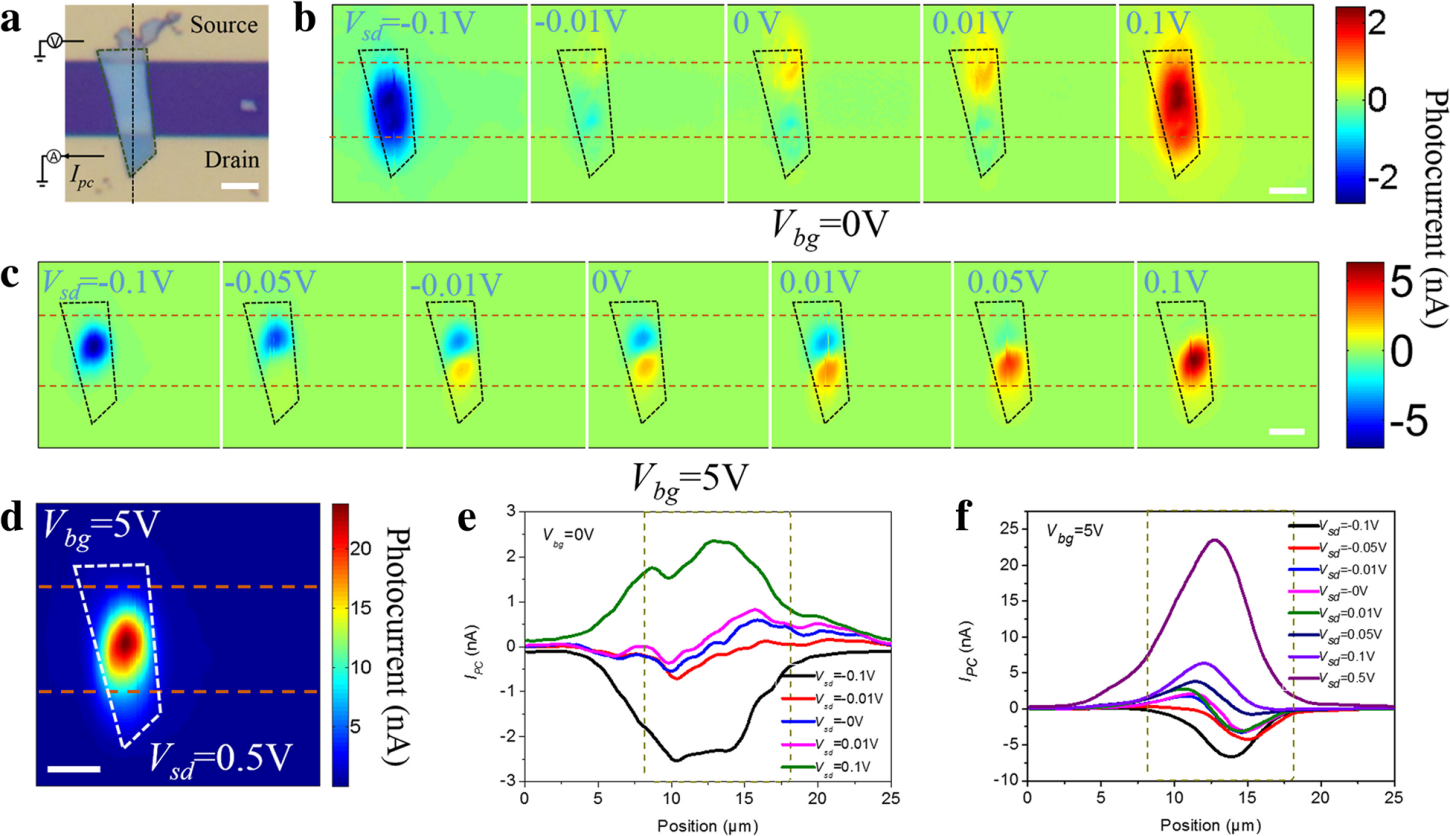
Spatial-resolved photocurrent images of D1 as a function of *V*_sd_. **a** The optical image together with the electrical setup. **b** Spatial-resolved photocurrent images at *V*_bg_ = 0 V and *V*_sd_ = − 0.1, 0.01, 0, 0.01, and 0.1 V, respectively. **c** Spatial-resolved photocurrent images at *V*_bg_ = 5 V and *V*_sd_ varies from − 0.1 to 0.1 V. **d** Spatial-resolved photocurrent images at *V*_bg_ = 5 V and *V*_sd_ = 0.5 V. **e** *I*_PC_ profile at *V*_bg_ = 0 V and **f** *I*_PC_ profile at *V*_bg_ = 5 V taken along dashed line in Fig. 4a. The scale bars are 5 μm in all figures

Figure 4 shows the spatial-resolved *I*_PC_ at different *V*_sd_ as *V*_bg_ = 0 and 5 V, respectively. Figure 4a shows the optical image, together with the electrical setup. *V*_sd_ is loaded on the source electrode, and *I*_PC_ is collected from the drain electrode. The current flowing from the source to drain electrode is positive. Figure 4b shows *I*_PC_ as a function of *V*_sd_ at *V*_bg_ = 0 V. When *V*_sd_ = 0, − 0.01, and 0.01 V, strong *I*_PC_ occurs in the vicinity of MoTe_2_/electrodes interface, then it moves toward the channel center as *V*_sd_ increases to 0.1 V. Similar trend is observed at *V*_bg_ = 5 V as *V*_sd_ increases as shown in Fig. 4c. Figure 4d shows a clear *I*_PC_ peak in the center of the device channel as *V*_sd_ increases to 0.5 V. *I*_PC_ profiles taken along the black dashed line in Fig. 4a are shown in Fig. 4e, f, which clearly show the *I*_PC_ variation trend as *V*_sd_ increases. They both indicate the maximum *I*_PC_ generated in the vicinity of contact interface in short-circuit condition or with small *V*_sd_ biased. When the biased voltage is increased, photocurrent peak moves toward the center of the device channel.

Based on these findings, we know that the potential step, formed in the vicinity of the electrodes/MoTe_2_ interface due to the doping of the MoTe_2_ by the metal contacts, dominates the separation of photoexcited electron-hole pairs in short-circuit condition or with small *V*_sd_ biased. Thus, *I*_PC_ at MoTe_2_-source is larger than that at MoTe_2_-drain due to the larger contact interface at MoTe_2_-source, and the net current is not zero, while the non-zero net current is smaller than *I*_sd_ at *V*_bg_ = − 5 and 0 V (in on-state), and larger than that at *V*_bg_ = 5 V (in off-state). Therefore, we observe clear *I*_SC_ at *V*_bg_ = 5 V as shown in Fig. 2b and Additional file 1: Figure S6(b)–(f). Therefore, both *I*_SC_ and the corresponding *V*_OC_ are the results of the potential step and asymmetric contact. Furthermore, we fabricate D2 sample with more asymmetric contact cross-section, as shown in Additional file 1: Figure S1, compared with D1. It shows a similar photovoltaic response, with *V*_OC_ as high as 150 mV when *V*_bg_ = 5 V and illumination laser wavelength is 637 nm. When the illumination wavelength varies to 830, 940, 1064, and 1312 nm, D2 shows a similar photovoltaic response at *V*_bg_ = 5 V (see Additional file 1: Figure S6 ). We also fabricate other four devices as shown in Additional file 1: Figure S8, they demonstrate the similar behavior to that has been shown in D1 and D2. These data further confirm that photovoltaic response of multi-layered MoTe_2_ phototransistor is a result from the asymmetric contact cross-section between MoTe_2_-source and MoTe_2_-drain electrodes.

**Fig. 5:**
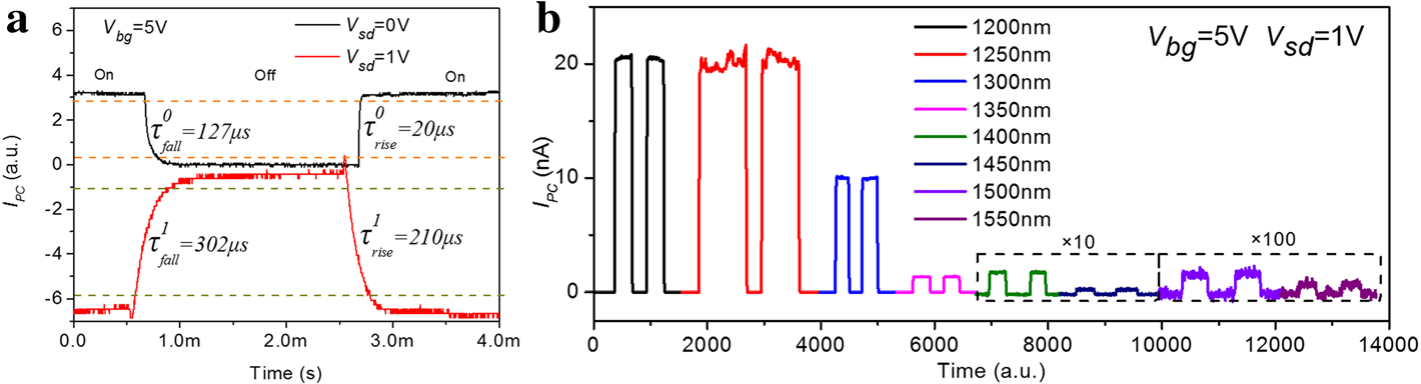
Photoresponse time and spectral range of multi-layered MoTe_2_ phototransistor. **a** Time-resolved photocurrent at *V*_bg_ = 5 V and *V*_sd_ = 0 V (black line) and 1 V (red line), respectively. **b** Photoresponse at different photoexcitation wavelengths

Finally, we test the photoresponse time and spectral range of multi-layered MoTe_2_ phototransistor. Figure 5a shows the time-resolved photocurrent at *V*_bg_ = 5 V and *V*_sd_ = 0 and 1 V, respectively, which are recorded using a current preamplifier and oscilloscope. The excitation laser is a square wave with 2 ms width at 637 nm wave-length. The currents collected under *V*_sd_ = 0 and 1 V show opposite direction, which is consistent with the data given in Fig. 2b, and is a result from the difference between *V*_OC_ and *V*_sd_. The rise time and fall time of photoresponse are defined as the time between 10 and 90% of the total photocurrent. As seen, the rise time $\left({\tau}_{\mathrm{rise}}^0\right)$ is 20 μs and fall time $\left(\ {\tau}_{\mathrm{fall}}^0\ \right)$ is 127 μs at *V*_sd_ = 0 V, and the rise time $\left({\tau}_{\mathrm{rise}}^1\right)$ is 210 μs and fall time $\left({\tau}_{\mathrm{fall}}^1\right)$ is 302 μs at *V*_sd_ = 1 V, which are both larger than that at *V*_sd_ = 0 V. This is because of the different mechanism of photocurrent generation. At *V*_sd_ = 0 V, the potential step-dominated photocurrent is generated in the vicinity of electrode/MoTe_2_ interface. At *V*_sd_ = 1 V, the photocurrent is generated in the device channel, and the photoexcited carriers have to go through the channel to arrive at the electrode, which takes longer time than the generation near the electrode/MoTe_2_ interface. Thus, the device shows longer photoresponse time at *V*_sd_ = 1 V than that at *V*_sd_ = 0 V. In addition to working at the visible band, a multi-layered MoTe_2_ phototransistor has photoresponse at the near-infrared band. Figure 5b shows that its photoresponse can be extended from 1200 to 1550 nm. Optical excitation, provided by SuperK EXTREME supercontinuum white light laser, is focused on the device channel center using a 20× objective lens with a spot diameter of 4.4 μm. The data indicate that multi-layered MoTe_2_ phototransistor can be used in the communication band.

## Conclusions

In summary, we have fabricated air-stable p-type multi-layered MoTe_2_ phototransistor with asymmetric contact form. Its photoresponse is investigated using scanning photocurrent at different gate and source-drain voltages, which helps to reveal the spatial potential profiles. The results indicate that potential step, formed in the vicinity of the electrodes/MoTe_2_ interface due to the doping of the MoTe_2_ by the metal contacts, plays an important role in separating photoexcited electron-hole pairs in short-circuit condition or with small *V*_sd_ biased. Net current is non-zero when potential step exists with asymmetric contact cross-section between MoTe_2_-source and MoTe_2_-drain electrodes. When biased voltage *V*_sd_ rises above potential step, *V*_sd_ dominates the separation of photoexcited electron-hole pairs, and *I*_PC_ peak appears in the center of the device channel. Moreover, MoTe_2_ phototransistor shows a faster response in short-circuit condition than that with higher biased *V*_sd_ within sub-millisecond, and its spectral range can be extended to the infrared end of 1550 nm.

## Methods/Experimental

Back-gated multi-layered MoTe_2_ phototransistors are fabricated in the following way. First, source, drain, and gate electrodes are patterned on 300-nm SiO_2_/p^+^-Si substrate using standard UV photolithography techniques, followed by selective etching of 300-nm SiO_2_ beneath the gate electrode and E-beam evaporation of a 5 nm/100 nm Cr/Au films. Second, the multi-layered MoTe_2_ sample is prepared on another 300-nm SiO_2_/p^+^-Si substrate by mechanical exfoliation of mm-size semiconducting 2H-MoTe_2_ single crystals, which is grown by chemical vapor transport using TeCl_4_ as the transport agent in a temperature gradient of 750 to 700 °C for 3 days. Finally, the prepared multi-layered MoTe_2_ sample is transferred onto patterned source-drain electrodes using polyvinyl alcohol (PVA) as a medium. PVA is dissolved in H_2_O and rinsed with isopropyl alcohol. Multi-layered MoTe_2_ samples are identified by an optical microscope, and the corresponding thickness is characterized using SPA-300HV atomic force microscopy (AFM). Raman signals are collected by a LabRAM HR Raman spectrometer with 514-nm wavelength laser excitation in the backscattering configuration using a 100 × objective.

Electrical characterization and photoresponse for 637-nm laser excitation are performed by combining Agilent B1500A semiconductor analyzer with Lakeshore probe station. The laser is illuminated onto the device using fiber and, the spot size is larger than 200 μm. Time-resolved photocurrent is recorded using a DL1211 current preamplifier and Keysight MSOX3024T oscilloscope. Spatial-resolved photocurrent is conducted using a home-made setup. The excitation laser is provided by SuperK EXTREME supercontinuum white light laser with an accessory of SuperK SELECT multi-line tunable filter to adjust the wavelength. The light is focused onto the device using a 20× objective lens and is chopped with SR570. The reflected light and the photocurrent are recorded with DL1211 current preamplifier and SR830 lock-in amplifier.

## Additional file

Additional file 1:
**Figure S1.** D2 properties. **Figure S2.** AFM image and corresponding height profile D1. **Figure S3.** Electric properties of D1. **Figure S4.** Electric properties of MoTe_2_ phototransistor with different thickness. **Figure S5.** Backgate-dependent and power-dependent photoresponse of D1. **Figure S6.** Photoresponse of D2 with different excitation wavelength. **Figure S7.** Normalized reflection in the vicinity of electrode. **Figure S8.** The photoresponse of other four multi-layered MoTe_2_ phototransistors. (DOCX 1617 kb)

## Abbreviations

2D: Two-dimensional
2H-MoTe_2_: 2H-type molybdenum ditelluride
AFM: Atomic force microscopy
FET: Field-effect transistor
*I*_PC_: Photocurrent
*I*_SC_: Short-circuit current
*I*_sd_: Source-drain current
PVA: Polyvinyl alcohol
TMDs: Transition metal dichalcogenides
*V*_bg_: Back-gate voltage
*V*_OC_: Open-circuit voltage
*V*_sd_: Source-drain voltage
*τ*_fall_: Fall time
*τ*_rise_: Rise time
