# Supplementary material for: Pronounced Photovoltaic Response from Multi-layered MoTe2 Phototransistor with Asymmetric Contact Form
Source: Nanoscale Res Lett. 2017 Nov 22;12:603. doi: 10.1186/s11671-017-2373-5 (PMC5700014; doi:10.1186/s11671-017-2373-5)
Supplement: Additional file 1: — Figure S1. D2 properties. Figure S2. AFM image and corresponding height profile D1. Figure S3. Electric properties of D1. Figure S4. Electric properties of MoTe2 phototransistor with different thickness. Figure S5. Backgate-dependent and power-dependent photoresponse of D1. Figure S6. Photoresponse of D2 with different excitation wavelength. Figure S7. Normalized reflection in the vicinity of electrode. Figure S8. The photoresponse of other four multi-layered MoTe2 phototransistors. (DOCX 1617 kb) [file 11671_2017_2373_MOESM1_ESM.docx]

Supporting Information

Pronounced Photovoltaic Response from Multi-layered MoTe_2_ Phototransistor with Asymmetric Contact Form

*Junku Liu^1*^, Nan Guo^1^,* *Xiaoyang Xiao^2^,* *Kenan Zhang^3^,* *Yi Jia^1^, Shuyun Zhou^3^,* *Yang Wu^2^, Qunqing Li^2^*, *Lin Xiao^1*^*

^1^*^.^* *Nanophotonics and Optoelectronics Research Center, Qian Xuesen Laboratory of Space Technology, China Academy of Space Technology, Beijing 100094, China*

^2.^ *State Key Laboratory of Low-Dimensional Quantum Physics, Department of Physics & Tsinghua-Foxconn Nanotechnology Research Center, Tsinghua University, Beijing 100084, China*

^3.^ *Department of Physics, Tsinghua University, Beijing 100084, China*

*Email addresses:*

*Junku Liu:* [*liujunku@qxslab.cn*](mailto:liujunku@qxslab.cn)

*Nan Guo: guonan@* *qxslab.cn*

*Xiaoyang Xiao:* *xiao-xy13@mails.tsinghua.edu.cn*

*Kenan Zhang: zhang-kn13@ mails.tsinghua.edu.cn*

*Yi Jia: jiayi@ qxslab.cn*

*Shuyun Zhou:* [*syzhou@mail.tsinghua.edu.cn*](mailto:syzhou@mail.tsinghua.edu.cn)

*Yang Wu: wuyang.thu@gmail.com*

*Qunqing Li:* [*qunqli@mail.tsinghua.edu.cn*](mailto:qunqli@mail.tsinghua.edu.cn)

*Lin Xiao:* [*xiaolin@qxslab.cn*](mailto:xiaolin@qxslab.cn)

*Corresponding authors:*

*J.L. (*[*liujunku@qxslab.cn*](mailto:liujunku@qxslab.cn)*) and L.X. (*[*xiaolin@qxslab.cn*](mailto:xiaolin@qxslab.cn)*)*


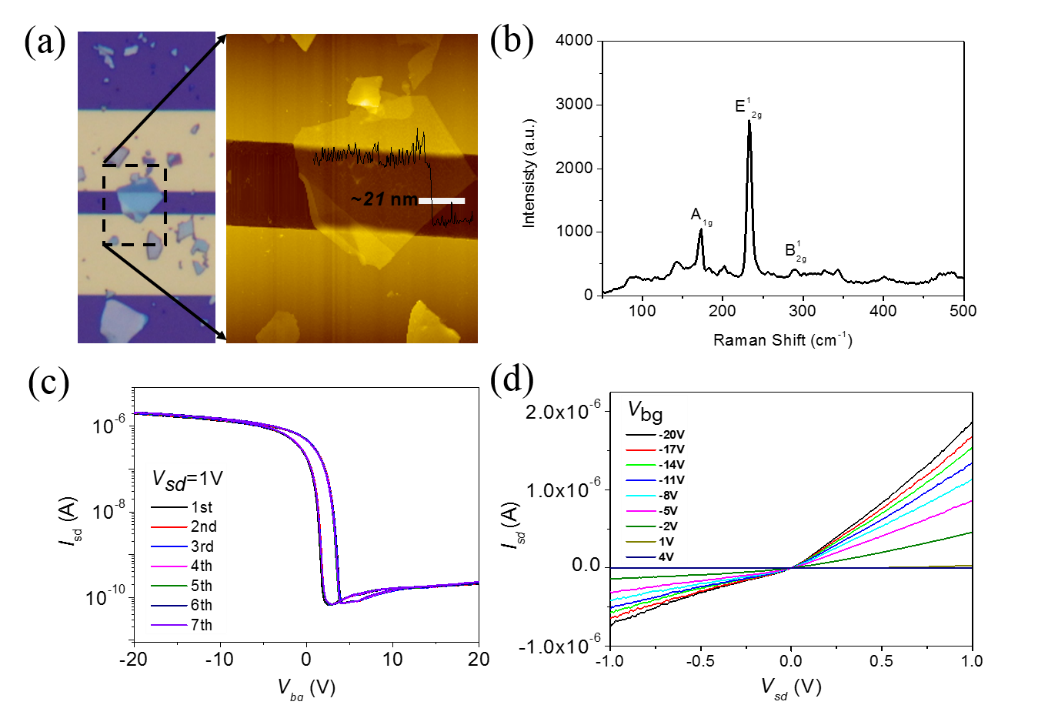


**Figure S1**. D2 properties. (a) Optical image (left) and AFM image (right) of D2, insert curve in AFM image indicates the thickness of MoTe_2_ is about 21nm. (b) Raman spectrum with three characteristic Raman-active modes of A_1g_ (172cm^-1^), E^1^_2g_ (233cm^-1^), and B^1^_2g_ (289cm^-1^) show the quality of MoTe_2_ in the channel. (c) Seven repeated transfer characteristics measured in ambient condition, which indicates multi-layered MoTe_2_ phototransistor has air-stable p-type conductance. Its on-off ratio is about 9×10^3^ as *V_sd_*=1V. When gate-voltage is swept from -20V to 20V and then back to -20V, the device shows small hysteresis. (d) Output characteristics as a function of back-gate voltage, which is asymmetric between positive and negative biased voltage.


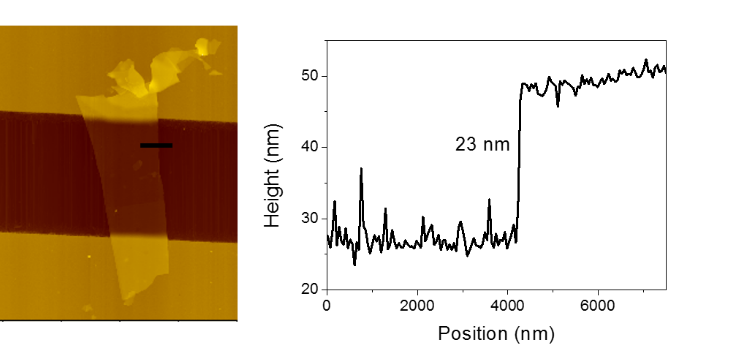


**Figure S2**. AFM image and corresponding height profile of multi-layered MoTe_2_ in D1.


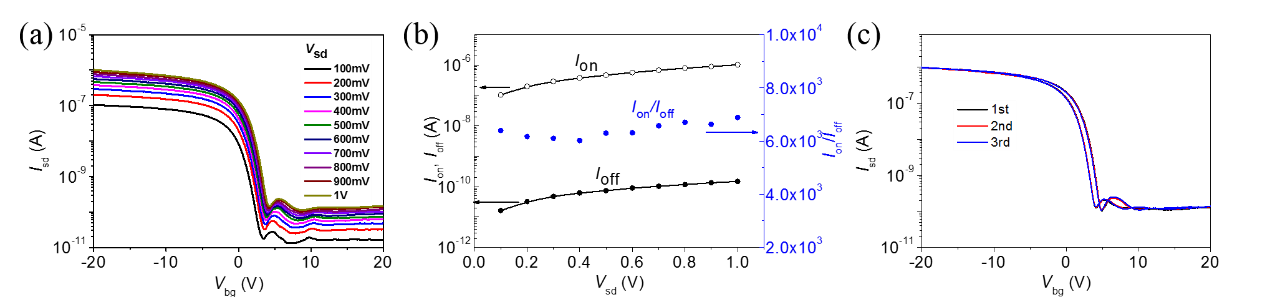


**Figure S3**. Electric properties of D1. (a) Transfer characteristics of D1 at different biased voltage. (b) On-current, Off-current and On-off ratio as a function of *V_sd_*. (c) Transfer characteristics as *V_bg_* is swept from -20V to 20V and back to -20V, which shows small hysteresis.

**
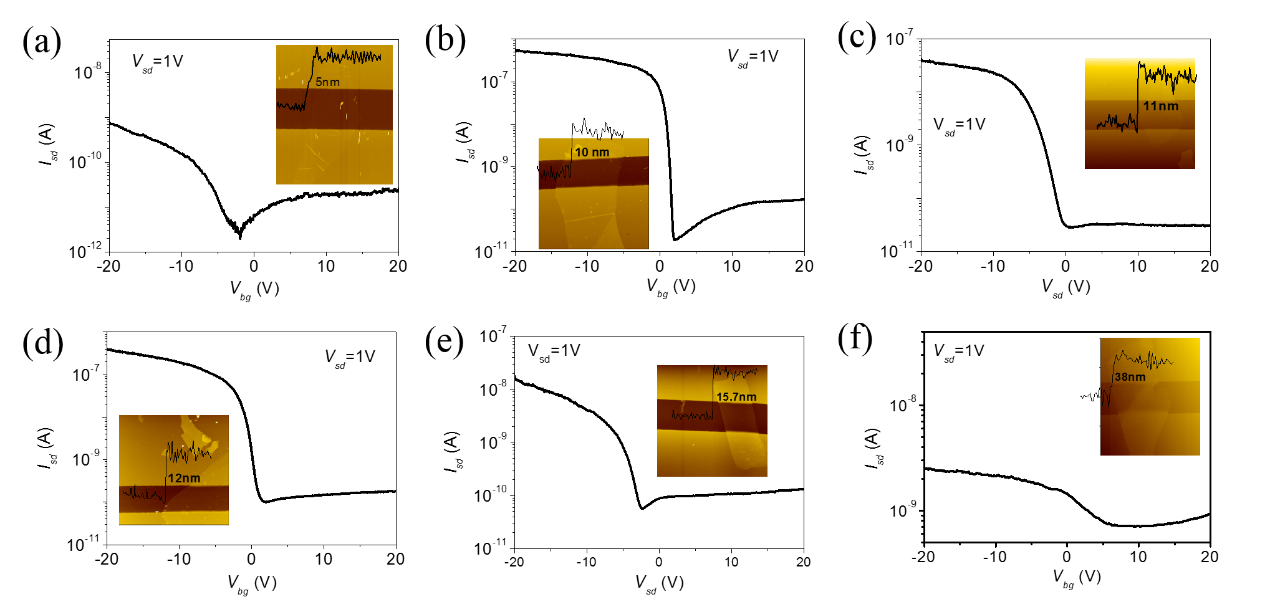
**

**Figure S4.** MoTe_2_ phototransistor with different thickness. (a)-(f) show the transfer characteristics of multi-layered MoTe_2_ phototransistor with thickness of 5nm, 10nm, 11nm, 12nm 15.7nm and 38nm, respectively. Inset image shows the AFM image and corresponding thickness.


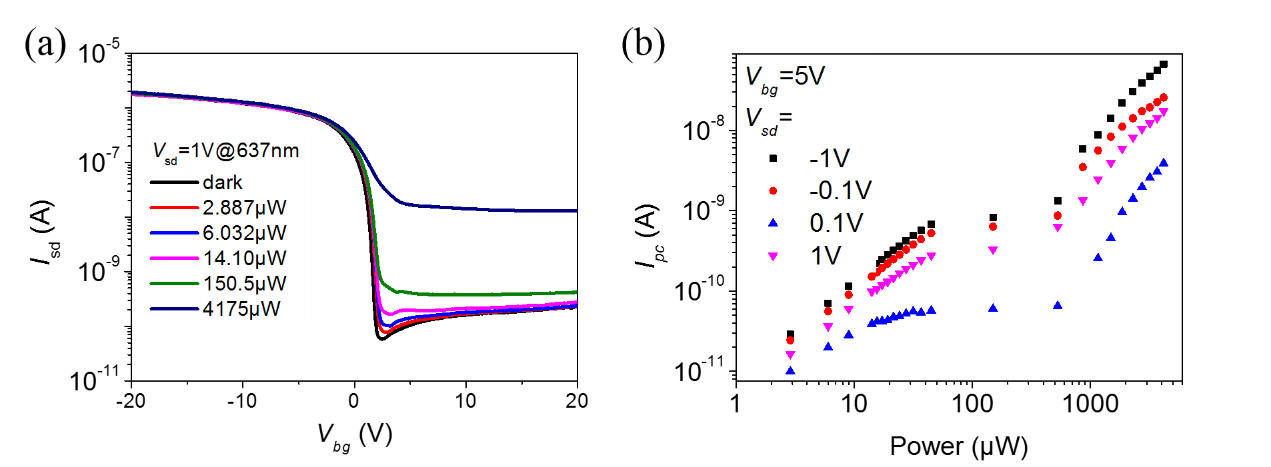


**Figure S5.** Backgate-dependent and power-dependent photoresponse of MoTe_2_ phototransistor illuminated by 637nm wavelength laser. (a) *I_sd_* vs. *V_bg_* curves at *V_sd_*=1V as illumination power increases from 0 W to 4175 μW. (b) Power-dependent photoresponse at different *V_sd_*.


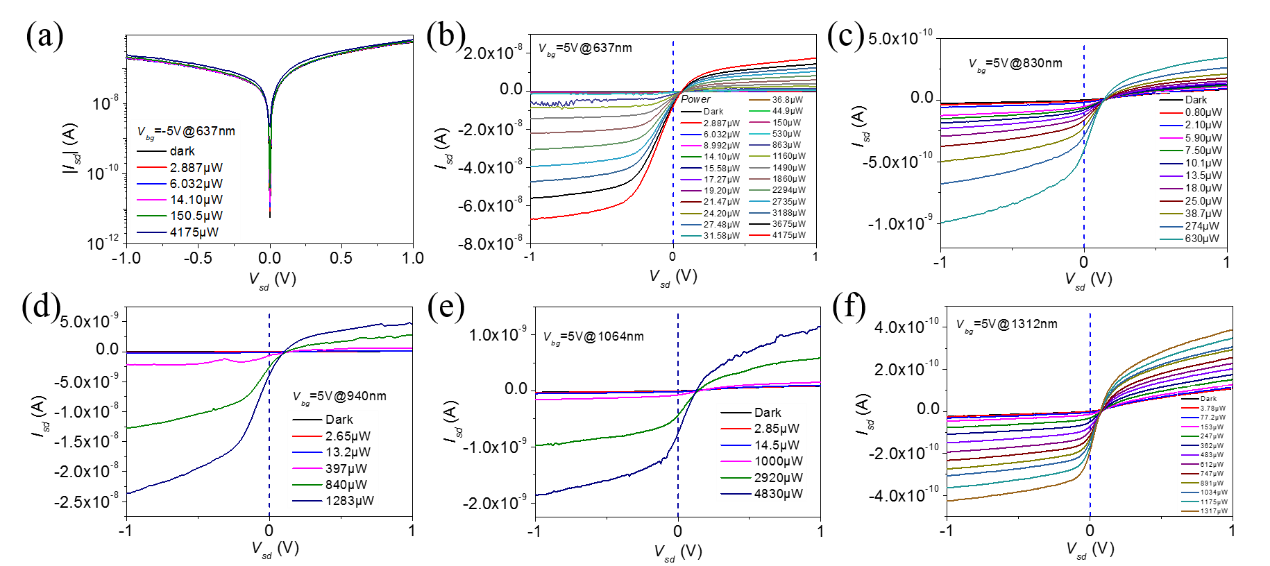


**Figure S6**. Photoresponse of D2. (a) Logarithmic plot of |*I_sd_*| vs. *V_sd_* at different illumination power for 637nm wavelength with *V_bg_* =-5V. *I_sd_* vs. *V_sd_* curve at *V_bg_* =5V at different illumination power for 637nm (b), 830nm (c), 940nm (d), 1064nm (e) and 1312nm (f). These data indicate the pronounced photovoltaic response at *V_bg_* =5V.


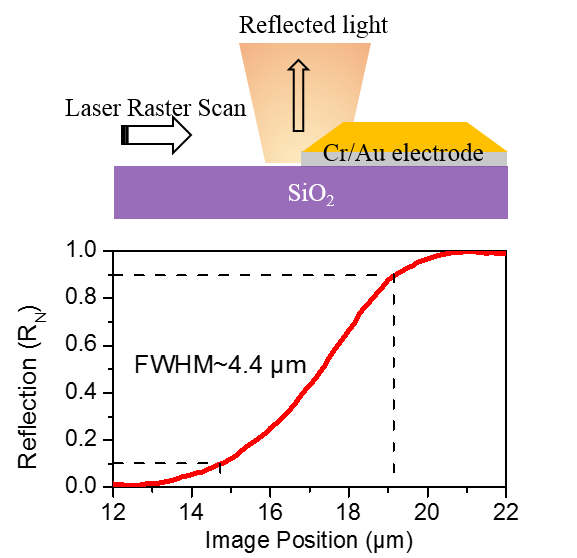


**Figure S7**. Normalized reflection in the vicinity of electrode. It is used to estimate the spot size of focused laser.


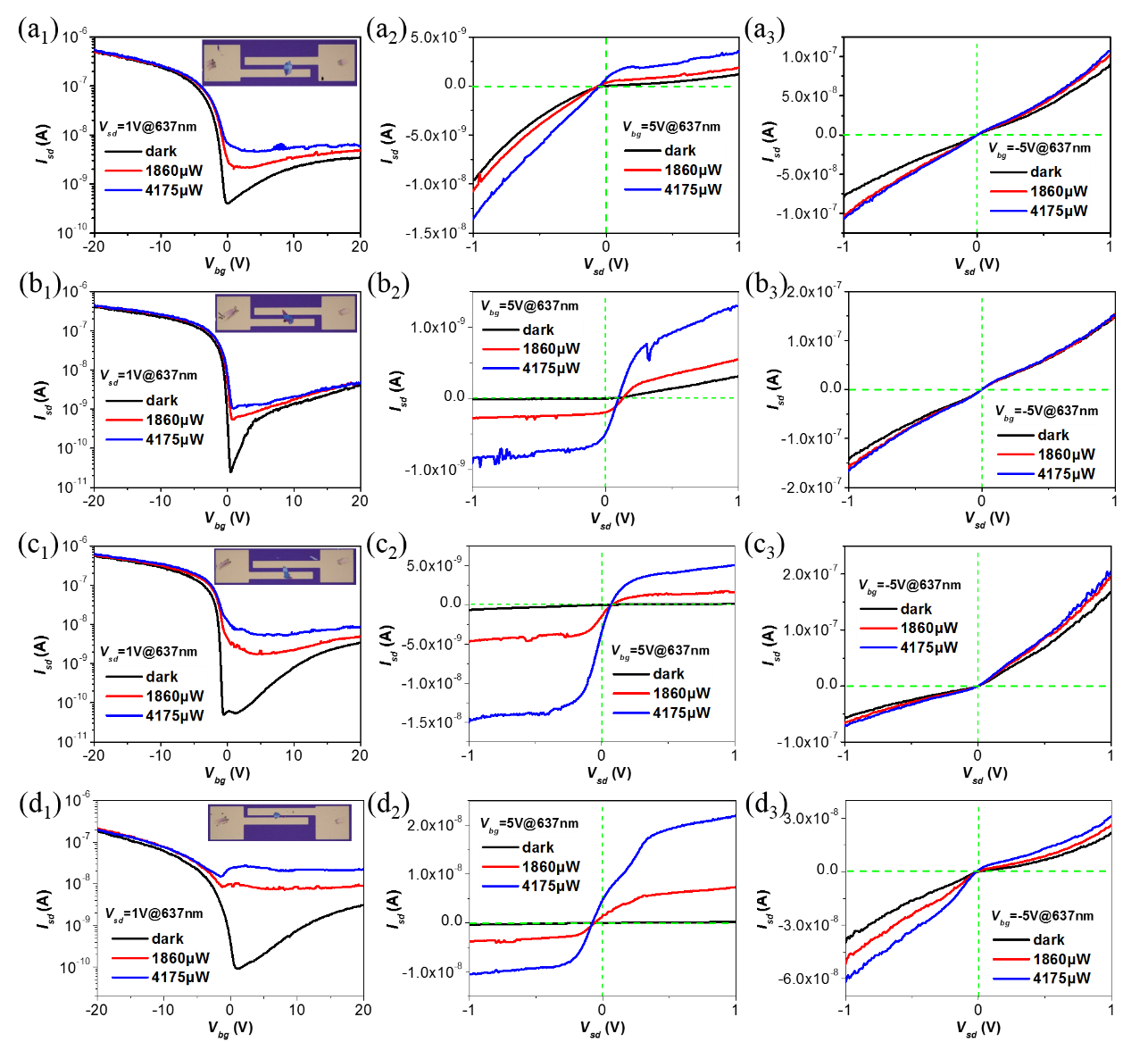


**Figure S8**. The photoresponse of other four multi-layered MoTe_2_ phototransistors illuminated by 637nm wavelength laser in ambient condition. (a_1_) ~ (a_3_) show *I_sd_* vs. *V_bg_* curves at *V_sd_*=1V, *I_sd_* vs. *V_sd_* curves at *V_bg_*=5V and *I_sd_* vs. *V_sd_* curves at *V_bg_*=-5V as illumination power increases, respectively, of 1st device. Inset image in figure (a_1_) is the optical image of 1st device. (b_1_) ~ (b_3_) show *I_sd_* vs. *V_bg_* curves at *V_sd_*=1V, *I_sd_* vs. *V_sd_* curves at *V_bg_*=5V and *I_sd_* vs. *V_sd_* curves at *V_bg_*=-5V as illumination power increases, respectively, of 2nd device. Inset image in figure (b_1_) is the optical image of 2nd device. (c_1_) ~ (c_3_) show *I_sd_* vs. *V_bg_* curves at *V_sd_*=1V, *I_sd_* vs. *V_sd_* curves at *V_bg_*=5V and *I_sd_* vs. *V_sd_* curves at *V_bg_*=-5V as illumination power increases, respectively, of 3rd device. Inset image in figure (c_1_) is the optical image of 3rd device. (d_1_) ~ (d_3_) show *I_sd_* vs. *V_bg_* curves at *V_sd_*=1V, *I_sd_* vs. *V_sd_* curves at *V_bg_*=5V and *I_sd_* vs. *V_sd_* curves at *V_bg_*=-5V as illumination power increases, respectively, of 4th device. Inset image in figure (d_1_) is the optical image of 4th device.
